# Transient receptor potential channel 6 knockdown prevents apoptosis of renal tubular epithelial cells upon oxidative stress via autophagy activation

**DOI:** 10.1038/s41419-018-1052-5

**Published:** 2018-10-03

**Authors:** Xin Hou, Haitao Xiao, Yanhong Zhang, Xixi Zeng, Mengjun Huang, Xiaoyun Chen, Lutz Birnbaumer, Yanhong Liao

**Affiliations:** 1 Department of Anatomy, Tongji Medical College, Huazhong University of Science and Technology, 430030 Wuhan, China; 2 Department of Anatomy, Medical College, Affiliated Hospital, Hebei University of Engineering, 056002 Handan, China; 3 Key Laboratory of Neurological Diseases of Ministry of Education, Tongji Medical College, Huazhong University of Science and Technology, 430030 Wuhan, China; 4 Department of Pathology, First Hospital of Wuhan, 430030 Wuhan, China; 5 Institute of Biomedical Research (BIOMED), Catholic University of Argentina, C1107AFF Buenos Aires, Argentina; 6 Neurobiology Laboratory, National Institute of Environmental Health Sciences, Research Triangle Park, Durham, NC 27709 USA

## Abstract

Reactive oxygen species (ROS) are generated under various pathological conditions such as renal ischemia/reperfusion (I/R) injury and provoke damage to multiple cellular organelles and processes. Overproduction of ROS causes oxidative stress and contributes to damages of renal proximal tubular cells (PTC), which are the main cause of the pathogenesis of renal I/R injury. Autophagy is a dynamic process that removes long-lived proteins and damaged organelles via lysosome-mediated degradation, which has an antioxidant effect that relieves oxidative stress. The canonical transient receptor potential channel 6 (TRPC6), a nonselective cation channel that allows passage of Ca^2+^, plays an important role in renal disease. Yet, the relationship between TRPC6 and autophagy, as well as their functions in renal oxidative stress injury, remains unclear. In this study, we found that oxidative stress triggered TRPC6-dependent Ca^2+^ influx in PTC to inhibit autophagy, thereby rendering cells more susceptible to death. We also demonstrated that TRPC6 knockout (TRPC6^-/-^) or inhibition by SAR7334, a TRPC6-selective inhibitor, increased autophagic flux and mitigated oxidative stress-induced apoptosis of PTC. The protective effects of TRPC6 ablation were prevented by autophagy inhibitors Chloroquine and Bafilomycin A1. Moreover, this study also shows that TRPC6 blockage promotes autophagic flux via inhibiting the PI3K/Akt/mTOR and ERK1/2 signaling pathways. This is the first evidence showing that TRPC6-mediated Ca^2+^ influx plays a novel role in suppressing cytoprotective autophagy triggered by oxidative stress in PTC, and it may become a novel therapeutic target for the treatment of renal oxidative stress injury in the future.

## Introduction

Renal ischemia/reperfusion (I/R) injury plays a pivotal role in renal transplantation and often results in early allograft dysfunction^1,2^. Reperfusion of blood flow into ischemic tissues induces a large generation of reactive oxygen species (ROS), including hydrogen peroxide (H_2_O_2_), superoxide anion (O^2-^), and hydroxyl radicals (·OH), further exacerbating tissue damages caused by ischemia. Because of the high metabolic rate, renal proximal tubular cells (PTC) suffer the most severe injury upon oxidative stress, which leads to cell damage and apoptosis^3–5^. Overproduction of ROS causes PTC damage, which is the main reason for the pathogenesis of renal oxidative stress injury. Suppression of ROS-induced PTC apoptosis is therefore critical for the treatment of renal injury upon oxidative stress.

Calcium (Ca^2+^) is an important second messenger implicated in diverse cellular functions, such as differentiation, gene expression, growth, and death^6,7^. Store-operated calcium entry (SOCE) is a ubiquitous Ca^2+^ entry mechanism, which induces sustained Ca^2+^ elevation and triggers Ca^2+^ overload under pathological stimuli. As components of store-operated Ca^2+^ channels (SOCs) and canonical transient receptor potential channels (TRPC) are nonselective Ca^2+^ permeable cation channels, which encompasses TRPC1–7^8,9^. Among these channels, TRPC6 is widely expressed in kidney cells, including tubular epithelial cells, podocytes, and glomerular mesangial cells and has been increasingly implicated in many forms of renal diseases^10–12^. Bioinformatics analysis by Shen et al.^13^ found that the expression of TRPC6 was upregulated upon renal I/R injury. On the other hand, recent studies have demonstrated that TRPC6 is a novel target of ROS in renal physiology and pathology^14,15^. However, whether TRPC6 plays a “pro-survival” or a “detrimental” role in renal oxidative stress injury remains controversial.

Autophagy is an important adaptive response that affects the function of many cells in both physiological and pathological conditions. During the process of renal I/R injury, autophagy is activated in PTC^16–18^. Additionally, ROS is produced and has been implicated as an upstream signal to induce autophagy^19,20^. Recently, despite the fact that autophagy can execute cell death in various conditions^21–23^, cumulative evidence supports a cytoprotective role of autophagy in renal oxidative stress injury^24–28^. Although ROS have been commonly accepted as an inducer of autophagy, how ROS regulates autophagy remains unclear. In recent years, the significant role of TRPCs in regulating autophagy has been demonstrated^29,30^, but the relationship between TRPC6 and autophagy is still poorly understood. Since both TRPC6 and autophagy play important roles in oxidative stress-induced renal injury, we investigated the physiological significance of ROS–TRPC6-mediated Ca^2+^ influx in autophagy regulation and its function in ROS-induced apoptosis of PTC.

Apoptosis and autophagy share many common regulatory molecules, such as Bcl-2 and the phosphatidylinositol 3-kinase (PI3K) /Akt signaling pathway^31^. It is well known that the PI3K/Akt pathway serves as a critical signaling axis in cell survival; however, strong evidence suggests that this pathway could also provide a pro-death signal^32,33^. The molecular mammalian target of rapamycin (mTOR) is a major downstream target of Akt. In addition, inhibition of the PI3K/Akt/mTOR pathway has been shown to initiate autophagy^32–35^. A growing body of evidence has suggested that activation of TRPC6 affects the Akt pathway^36,37^. The Ras⁄Raf⁄ERK signaling pathway also plays a crucial role in autophagy regulation. Schnellmann et al.^38^ showed that the ERK1/2 pathway participated in H_2_O_2_-induced PTC apoptosis by inducing mitochondrial cytochrome c release and activating caspase-3. Mograbi et al.^39,40^ showed in their earlier studies that sustained activation of the ERK1/2 pathway disrupted the maturation of autophagosomes into functional autolysosomes and inhibited autophagy. Accordingly, this study aims to explore the effect of TRPC6 in regulating the PI3K/Akt and ERK signaling pathways in response to oxidative stress and its impact on autophagy.

In this study, we aimed at identifying the role of TRPC6-mediated SOCE in H_2_O_2_-induced autophagy and apoptosis in PTC. Our results suggest that Ca^2+^ entry via TRPC6 has an inhibitory effect on H_2_O_2_-mediated autophagy via activating the PI3K/Akt/mTOR and Ras/Raf/ERK pathways. In addition, we found that TRPC6 knockout or inhibition by SAR7334 increases autophagic flux and partially decreases H_2_O_2_-induced apoptosis of PTC. Furthermore, we show that autophagy blockage prevents the protective effect of TRPC6 inhibition or knockout on H_2_O_2_-induced PTC apoptosis. In conclusion, we demonstrated that oxidative stress treatment increases TRPC6 expression and triggers Ca^2+^ influx via TRPC6-mediated SOCE to activate Akt and ERK pathways to inhibit autophagy, which renders cells more vulnerable to death. Accordingly, TRPC6 inhibition prevents PTC apoptosis upon oxidative stress partially via autophagy activation.

## Results

### Oxidative stress increases TRPC6 expression and triggers Ca2+ influx via TRPC6-mediated SOCE

**Fig. 1:**
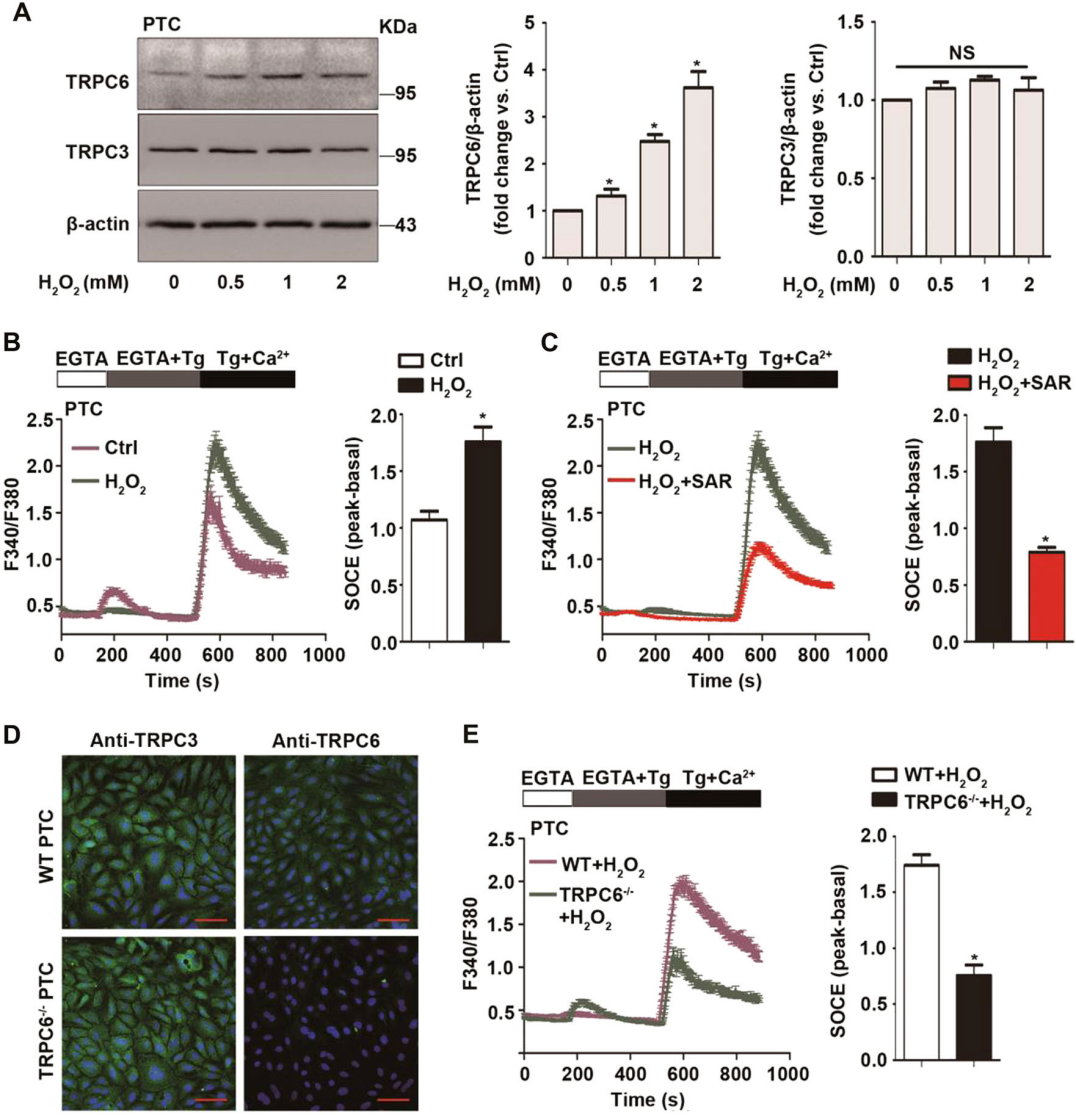
Oxidative stress increases TRPC6 expression and triggers Ca^2+^ influx via TRPC6-mediated store-operated Ca2+ entry (SOCE). **a** Representative western blot images of TRPC6 and TRPC3 in primary PTC after treatment with different concentrations of H_2_O_2_ for 12 h. Data are expressed as mean ± SEM, *n* = 3; NS indicates not significant, *P < 0.05. **b** Representative traces showing the Thapsigargin (Tg)-evoked transient increase in [Ca^2+^]_i_ (SOCE) after treatment with 0.5 mM H_2_O_2_ for 30 min or left untreated. Quantification of peak SOCE values are expressed as mean ± SEM, *n* = 3 (40–60 cells for each independent experiment); *P < 0.05. **c** Representative traces showing the Tg-evoked SOCE after treatment with H_2_O_2_ in the presence and absence of TRPC6 inhibitor SAR7334 (100 nM). Quantification of peak SOCE values are expressed as mean ± SEM, *n* = 3 (40–60 cells per experiment); *P < 0.05. **d** Immunohistochemistry analysis of the TRPC6 and TRPC3 expression in PTC isolated from WT and TRPC6^-/-^ mice, Scale Bar = 20 μm. **e** Representative traces showing the Tg-evoked SOCE in PTC isolated from WT and TRPC6^-/-^ mice after treatment with H_2_O_2_. Quantification of peak SOCE values are expressed as mean ± SEM, *n* = 3 (40–60 cells per experiment); *P < 0.05

Primary PTC were stimulated with different concentration of H_2_O_2_ (Fig. 1a) or tert-butyl hydroperoxide (t-BOOH) (Fig. S1a) for 12 h. It has been previously reported that TRPC3, TRPC6, and TRPC7 are homologous and always work synergistically in various pathological processes^41,42^. Since the kidney lacks TRPC7 expression^43^, we tested the expression of TRPC3 and TRPC6 in H_2_O_2_-treated cells. We observed that oxidative stress enhanced TRPC6 but not TRPC3 expression in PTC compared with the control group. These results are consistent with the previous results of Shen et al.^13^.

TRPCs have functional significance in cellular Ca^2+^ signaling. They may function as a store-operated Ca^2+^ channel (SOC) activated by depletion of intracellular Ca^2+^ stores^44^ or as a receptor-operated Ca^2+^ channel (ROC) activated by G protein-coupled and receptor tyrosine kinase signaling pathways^45^. As SOCE is the principal means of Ca^2+^ influx in nonexcitable cells, including PTC, we evaluated the function of TRPC6 in Thapsigargin (Tg) (a sarcoplasmic reticulum Ca^2+^ ATPase inhibitor)-triggered SOCE in primary PTC. Calcium imaging results showed that H_2_O_2_ treatment increased SOCE, which was abolished by pretreatment with the specific TRPC6 inhibitor SAR7334 (Fig. 1b, c). To confirm the function of TRPC6 in SOCE of PTC, TRPC6^-/-^ mice were used, and immunohistochemistry confirmed that PTC from TRPC6^-/-^ mice lack the TRPC6 isoforms and had normal TRPC3 expression compared with PTC from WT mice (Fig. 1d). Calcium imaging showed that the SOCE peak of TRPC6^-/-^ PTC was much smaller than that of WT PTC (Fig. S2). More importantly, H_2_O_2_-triggered SOCE was obviously reduced in TRPC6^-/-^ PTC (Fig. 1e). Given the data showing that H_2_O_2_ treatment increases TRPC6 expression, this could prove that increased TRPC6 protein expression leads to more functional TRPC6 channels and increased SOCE.

### TRPC6 knockout prevents H_2_O_2_-mediated autophagy inhibition

**Fig. 2:**
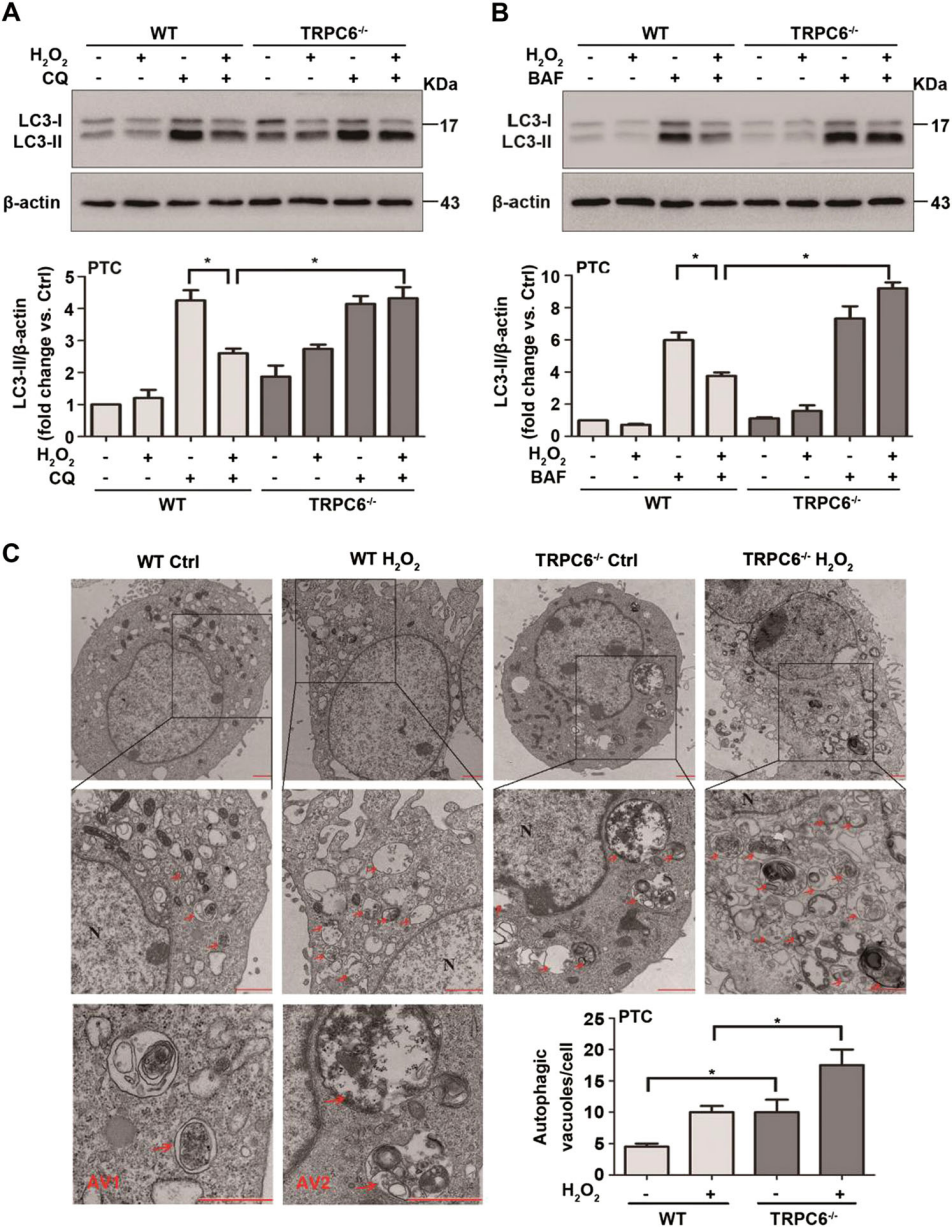
TRPC6 knockout prevents H_2_O_2_-mediated autophagy inhibition. **a, b** Representative western blot images of LC3 (LC3I and LC3II) in primary PTC were isolated from WT and TRPC6^-/-^ mice after treatment with H_2_O_2_ (0.5 mM 12 h) in the presence and absence of the autophagy inhibitors chloroquine (CQ) (25 μM) and bafilomycin A1 (BAF) (20 nM). Relative quantification of LC3II are expressed as mean ± SEM, *n* = 3; *P < 0.05. **c** Ultrastructural images of autophagic vacuoles in H_2_O_2_ (0.5 mM 6 h)-treated and nontreated cells were detected by transmission electron microscopy. Arrow autophagic vacuoles, N nucleus, AV1 autophagosomes, AV2 autolysosomes; Scale Bar = 1 μm. Bar diagram is representing the number of autophagic vacuoles in different groups. Data are expressed as mean ± SEM, *n* = 3 (20–30 cells per experiment); *P < 0.05

To explore the function of TRPC6 in oxidative stress-mediated autophagy regulation, primary PTC of WT and TRPC6^-/-^ mice were treated with 0.5 mM H_2_O_2_ for 12 h to mimic oxidative stress in vitro. The microtubule-associated protein 1 light-chain 3 (LC3)-II is the most widely monitored autophagy-related protein^46^. Primary PTC exhibited rapid formation of autophagosomes and LC3-II expression in response to oxidative stress. However, prolonged (12 h) H_2_O_2_ or t-BOOH treatment attenuated LC3-II expression (Fig. S1b, c) and was accompanied by a significant increase in TRPC6 expression and apoptosis. To assess autophagic flux, accumulation of LC3-II was obtained by interrupting the autophagosome–lysosome fusion step, by specifically inhibiting the V-ATPase with bafilomycin A1 (BAF) or by raising the lysosomal pH by the addition of chloroquine (CQ). As expected, it showed a remarkable increase in LC3-II levels after CQ or BAF treatment (Fig. 2a, b). It is worth noting that H_2_O_2_ treatment markedly decreased LC3-II levels induced by CQ and BAF, indicating an impaired autophagic flux in H_2_O_2_-treated cells. Conversely, compared with the WT PTC, H_2_O_2_ treatment in TRPC6^-/-^ PTC markedly increased the LC3-II levels induced by CQ and BAF (Fig. 2a, b). These data indicate that H_2_O_2_ triggers Ca^2+^ influx via TRPC6 to inhibit autophagic flux.

To confirm this result, ultrastructural images of autophagic vacuoles in PTC from WT and TRPC6^-/-^ mice upon H_2_O_2_ treatment were inspected by electron microscopy. After H_2_O_2_ treatment (0.5 mM, 6 h), the autophagic vacuoles were increased. Interestingly, autophagic vacuoles were increased in both the H_2_O_2_-treated and untreated PTC of TRPC6^-/-^ mice. Moreover, we found that PTC from TRPC6^-/-^ mice had more autophagosomes and autolysosomes than PTC from WT mice (Fig. 2c), which indicates a higher level of autophagic flux in TRPC6^-/-^ PTC. These phenomena suggest that TRPC6 plays an important role in autophagy regulation.

### TRPC6 inhibition promotes autophagic flux in HK-2 cells

**Fig. 3:**
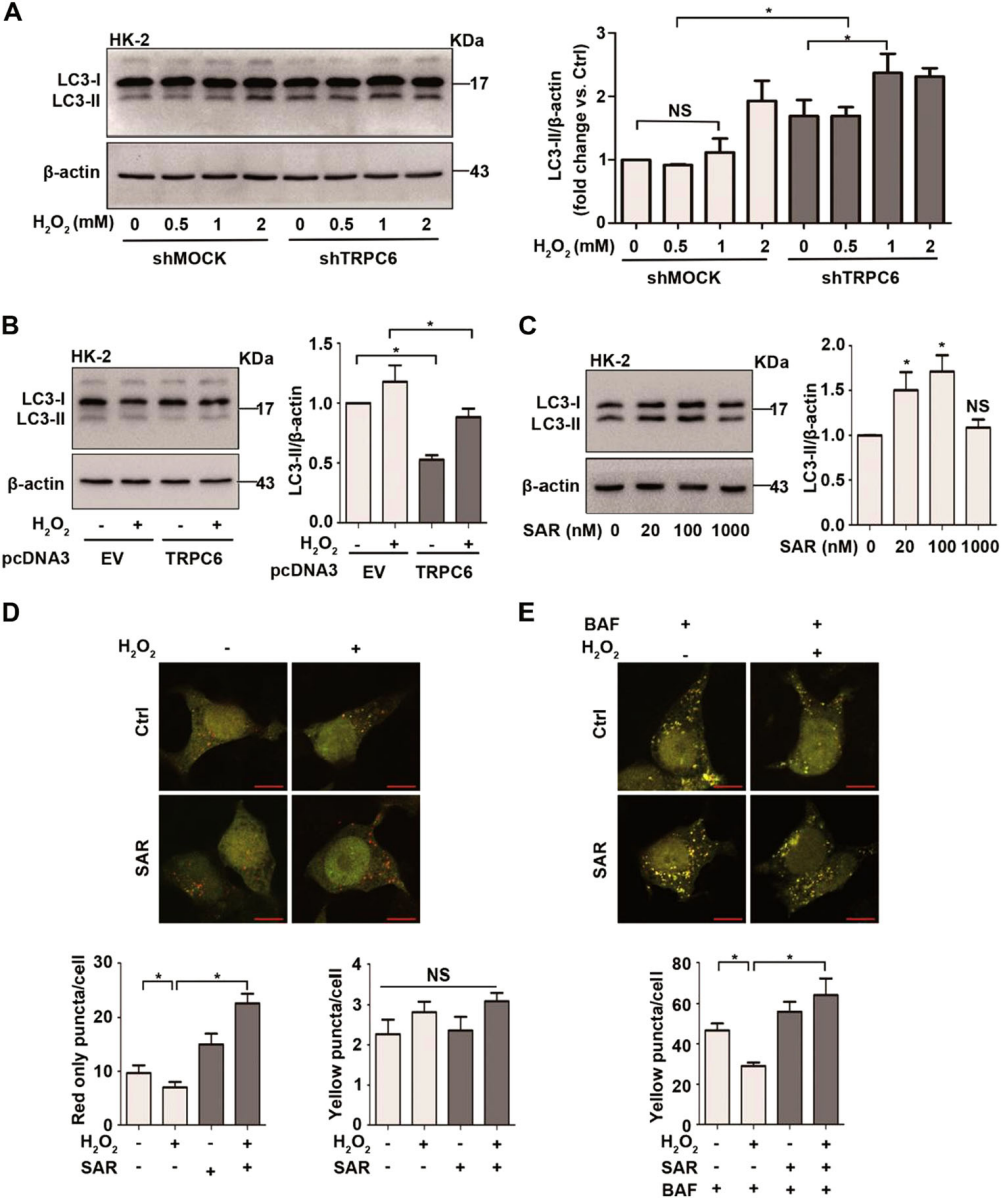
TRPC6 inhibition promotes autophagic flux in HK-2 cells **a** HK-2 cells were transfected with shTRPC6 or shMOCK plasmid for 48 h before treatment with different concentrations of H_2_O_2_ for 12 h. Representative western blot images and the relative quantification of LC3-II are shown. **b** HK-2 cells were transfected with pcDNA3-TRPC6 or pcDNA3-EV plasmid for 48 h before treatment with 0.5 mM H_2_O_2_ for 12 h. Representative western blot images and the relative quantification of LC3-II are shown. **c** HK-2 cells were treated with different concentrations of SAR7334 for 12 h. Representative western blot images and the relative quantification of LC3-II are shown. All data are expressed as mean ± SEM, *n* = 3; NS indicates not significant, *P < 0.05. **d, e** HK-2 cells were transfected with tandem mRFP-GFP-LC3 plasmid for 48 h and then exposed to 0.5 mM H_2_O_2_ for 12 h in the absence and presence of SAR (100 nM) and BAF (20 nM). Images were captured with laser confocal scanning microscopy (LCSM), Scale Bar = 20 μm. Bar graphs show the quantitative analysis of red and yellow puncta in images. Data are expressed as mean ± SEM, *n* = 3 (50–60 cells per experiment); NS indicates not significant, *P < 0.05

ShTRPC6 and pcDNA3-TRPC6 plasmids were used to investigate the relationship between TRPC6 and autophagy. After sh-TRPC6 lentivirus infection, the mRNA and protein expression of TRPC6 were downregulated (Fig. S3a). Semi-quantitative immunoblotting demonstrated that silencing TRPC6 in HK-2 cells increased the expression of LC3-II compared with shMOCK infected cells (Fig. 3a). These results suggest that TRPC6 knockdown promotes autophagic flux upon H_2_O_2_ treatment. To confirm the inhibitory effect of TRPC6 on autophagy, we used a pcDNA3-TRPC6 plasmid to overexpress TRPC6 in HK-2 cells, and the mRNA and protein expression of TRPC6 were upregulated (Fig. S3b). The overexpression of TRPC6 inhibited the expression of LC3-II compared with pcDNA3-EV transfected cells (Fig. 3b). These results suggest that silencing or overexpressing TRPC6 influences not only basal but also H_2_O_2_-induced autophagy.

To further confirm the role of TRPC6-triggered Ca^2+^ entry in oxidative stress-mediated autophagy inhibition, SAR7334, a potent and specific TRPC6 inhibitor^47^ was used. IC_50_ values are 9.5, 226, and 282 nM for TRPC6, TRPC7, and TRPC3-mediated Ca^2+^ influx, respectively. In the present study, we found that the expression of LC3-II was significantly increased in primary PTC after low concentrations of SAR7334 (20–100 nM) treatment for 12 h (Fig. 3c). To assess the function of SAR7334 on H_2_O_2_-mediated autophagic flux, we transfected HK-2 cells with a construct expressing LC3 tagged in tandem with monomeric red fluorescent protein and green fluorescent protein (mRFP-GFP) to examine the autophagosome maturation process. In merged images, the yellow and red puncta represent autophagosomes and autolysosomes, respectively, because mRFP, but not GFP, retains fluorescence in the acidic environment of lysosomes^48^. The results showed that 0.5 mM H_2_O_2_ treatment for 12 h markedly decreased the red LC3-II and yellow LC3-II puncta induced by BAF (Fig. 3d, e). After exposure to 100 nM SAR7334 for 12 h, the red puncta were increased (Fig. 3d). After treatment with H_2_O_2_ and BAF, an increase of yellow puncta was observed in SAR7334 pretreated cells, indicating that SAR7334 promotes autophagic flux (Fig. 3e). These results demonstrate that TRPC6 blockage restored H_2_O_2_-induced autophagy inhibition in PTC.

### TRPC6 inhibition mitigates H_2_O_2_-induced apoptosis in primary PTC

**Fig. 4:**
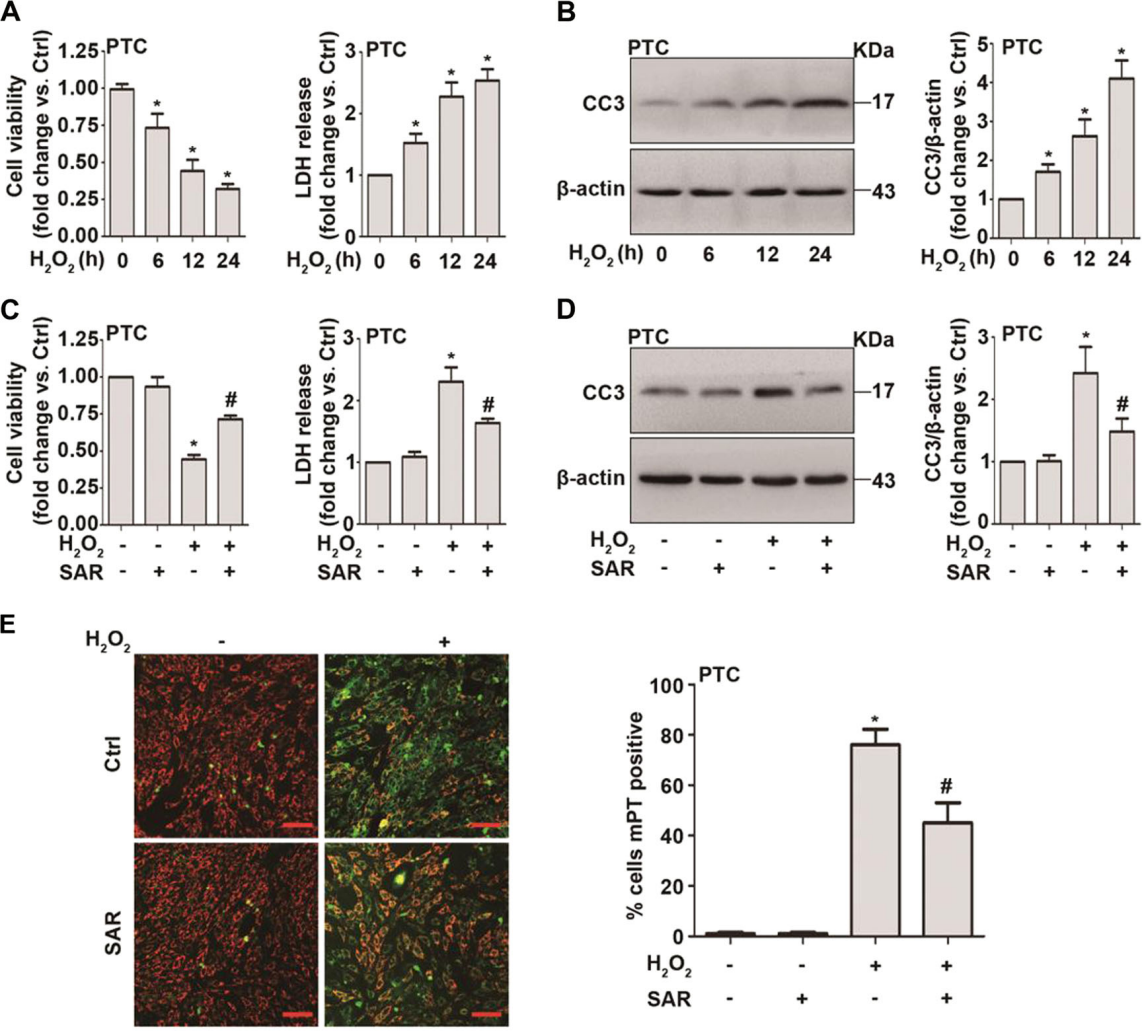
TRPC6 inhibition mitigates H_2_O_2_-induced apoptosis in primary PTC. **a** PTC isolated from WT mice were treated with H_2_O_2_ (0.5 mM) for different times. The viability and LDH release of PTC was measured. All data are expressed as mean ± SEM, *n* = 6; *P < 0.05. **b** Representative western blot images and the relative quantification of cleaved caspase-3 (CC3). Data are expressed as mean ± SEM, *n* = 4; *P < 0.05. **c** PTC isolated from WT mice were treated with H_2_O_2_ (0.5 mM) in the absence and presence of SAR7334 (100 nM) for 12 h. The viability and LDH release of PTC was measured. All data are expressed as mean ± SEM, *n* = 3; *P < 0.05 vs. control, ^#^*P* < 0.05 vs. the H_2_O_2_ group. **d** Representative western blot images of CC3 after treatment with H_2_O_2_ (0.5 mM) in the absence and presence of SAR7334 (100 nM) for 12 h. Bar graph is showing the relative quantification of CC3. Data are expressed as mean ± SEM, *n* = 3; *P < 0.05 vs. control, ^#^*P* < 0.05 vs. the H_2_O_2_ group. **e** PTC were treated with H_2_O_2_ (0.5 mM) in the absence and presence of SAR7334 (100 nM) for 12 h. Mitochondrial membrane potential was measured using JC-1 dye. Bar diagram is showing the number of mPT (mitochondrial permeability transition)-positive cells upon H_2_O_2_ treatment. Data are expressed as mean ± SEM, *n* = 3; Scale Bar = 50 μm, *P < 0.05 vs. control, ^#^*P* < 0.05 vs. the H_2_O_2_ group

Primary PTC were stimulated with H_2_O_2_ (0.5 mM) for different times. CCK-8 assays and LDH tests showed that H_2_O_2_ treatment decreased cell viability and increased LDH release in a time-dependent manner (Fig. 4a). Western blot results showed that after H_2_O_2_ treatment, the level of the apoptosis marker, cleaved caspase-3 (CC3, an activated form of caspase-3), increased dramatically (Fig. 4b).

Whether TRPC6 has a “pro-survival” or a “detrimental” role in H_2_O_2_-induced injury remains unknown. The CCK-8 assay and LDH detection showed that SAR7334 treatment partially improved cell viability and decreased LDH release upon H_2_O_2_ treatment (Fig. 4c). Importantly, after SAR7334 treatment, the activation of caspase-3 induced by H_2_O_2_ was markedly reversed (Fig. 4d). The mitochondrial permeability transition (mPT), which results from the assembly of the mitochondrial permeability transition pore (mPTP) and the collapse of the mitochondrial membrane potential (ψm), is one of the hallmarks of oxidative stress injury. As further evidence, the collapse of the mitochondrial membrane potential caused by H_2_O_2_, which was detected by a tetrechloro-tetraethylbenzimidazol carbocyanine iodide (JC-1) reporter dye, was partially rescued by SAR7334 pretreatment (Fig. 4e). The mPT-positive PTC decreased dramatically by SAR7334 (Fig. 4e). All of these results show that TRPC6 inhibition has a protective effect in H_2_O_2_-treated PTC.

### TRPC6 knockout attenuates oxidative stress-induced cell apoptosis

**Fig. 5:**
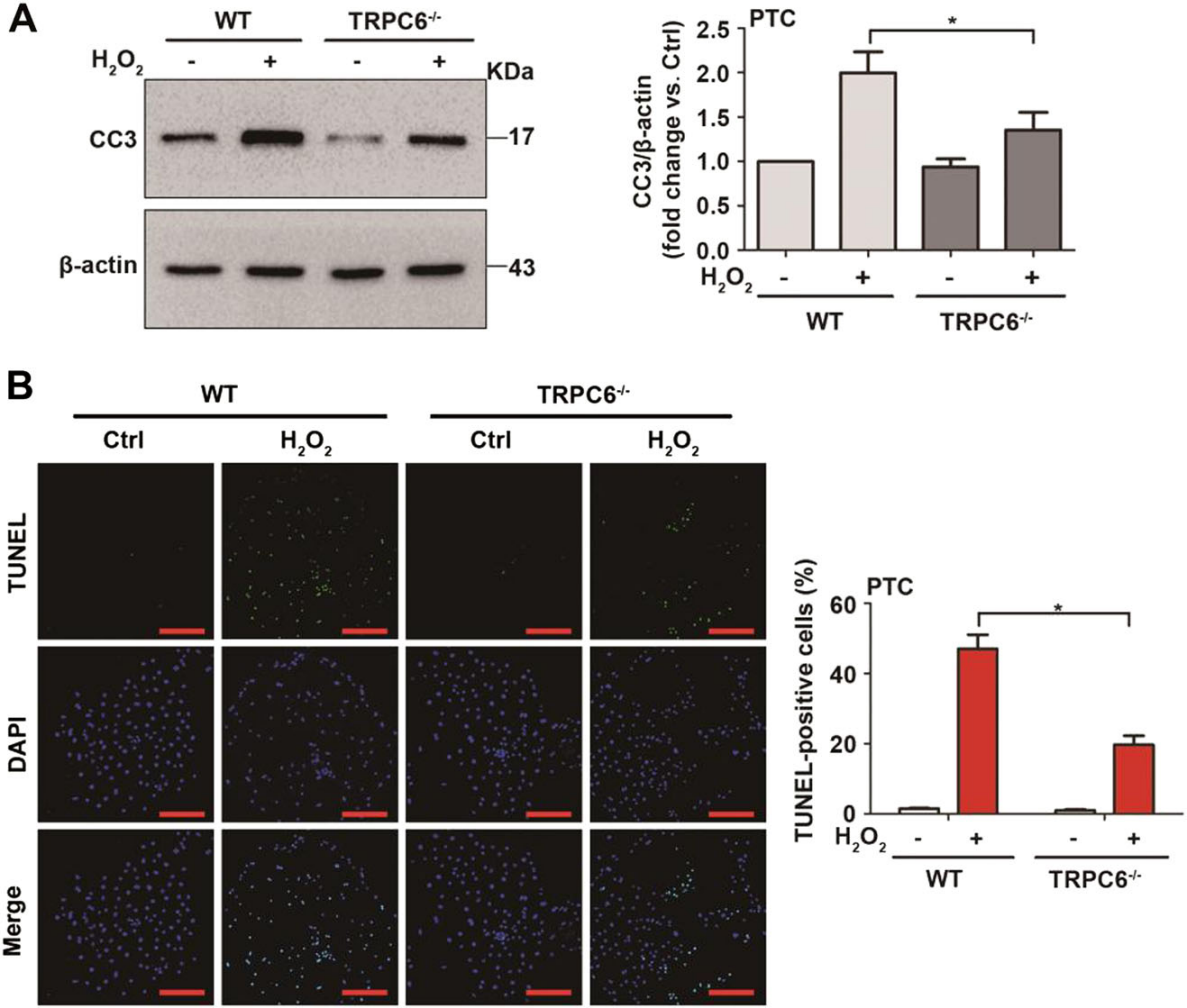
TRPC6 knockout attenuates oxidative stress-induced cell apoptosis. Primary PTC from WT and TRPC6^-/-^ mice were divided into different groups and treated with H_2_O_2_ (0.5 mM) for 12 h. **a** Representative western blot images and the relative quantification of cleaved caspase-3 (CC3). Data are expressed as mean ± SEM, *n* = 3; *P < 0.05. **b** Representative TUNEL staining of PTC in each group. Scale Bar = 50 μm. Bar graph is showing the quantification of TUNEL-positive cells. Data are expressed as mean ± SEM, *n* = 6; *P < 0.05

To further clarify the role of TRPC6-mediated Ca^2+^ signaling in oxidative stress-induced PTC injury, TRPC6^-/-^ mice were used. As expected, we found that the increased level of CC3 upon H_2_O_2_ (Fig. 5a) and t-BOOH (Fig. S1d) treatment was dramatically prevented in TRPC6^-/-^ PTC. Similarly, as shown by the TUNEL assay, TRPC6^-/-^ mice had a decreased proportion of cells undergoing apoptosis upon H_2_O_2_ treatment (Fig. 5b). These results indicate that TRPC6 knockout alleviates oxidative stress-induced apoptosis of PTC.

### Autophagy blockage prevents the protective effect of TRPC6 knockout

**Fig. 6:**
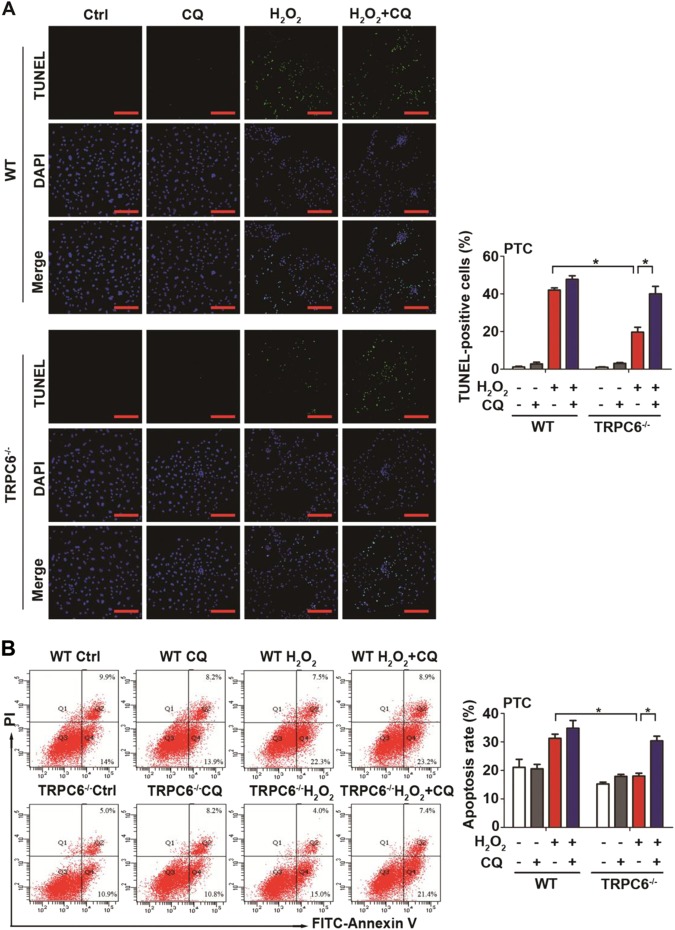
Blockage of autophagy prevents the protective effect of TRPC6 knockout. PTC isolated from WT or TRPC6^-/-^ mice were divided into eight different groups and treated with H_2_O_2_ (0.5 mM) in the absence and presence of CQ (25 μM) for 12 h. **a** Representative TUNEL staining of PTC in each group, Scale Bar = 50 μm. Bar graph is showing the quantification of TUNEL-positive cells. Data are expressed as mean ± SEM, *n* = 6; *P < 0.05. **b** Representative flow cytometric assessment of apoptosis via double-staining with Annexin V-FITC and PI. Bar diagram is showing the apoptosis rates of different groups. Data are expressed as mean ± SEM, *n* = 3; *P < 0.05

The autophagy inhibitor, CQ, was used to confirm whether the protective effect of TRPC6 inhibition was due to the activation of autophagy. As shown by the TUNEL assay, TRPC6^-/-^ mice had a decreased proportion of cells undergoing apoptosis upon H_2_O_2_ treatment. Moreover, the addition of CQ dramatically increased the apoptotic ratio in TRPC6^-/-^ PTC as compared with WT counterparts (Fig. 6a). Likewise, the flow cytometry results showed that the addition of CQ caused significant cell apoptosis and counteracted the protective effect of TRPC6 knockout (Fig. 6b). Altogether, these results indicate that TRPC6 knockout alleviates oxidative stress-induced apoptosis by promoting autophagic flux.

### TRPC6 knockout activates autophagy via negatively regulating the PI3K/Akt/mTOR and ERK1/2 signaling pathways

**Fig. 7:**
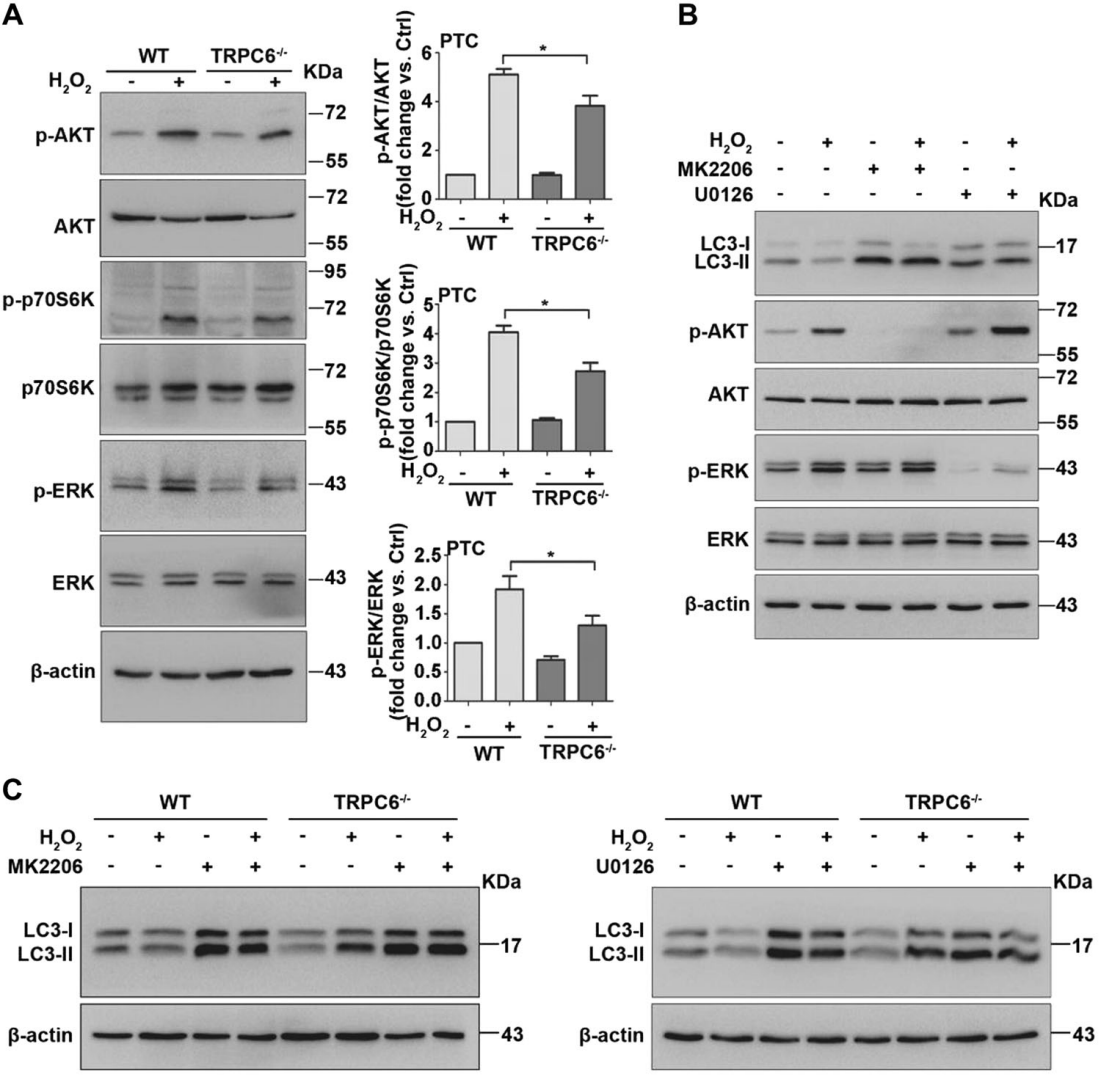
TRPC6 inhibits autophagic flux via positively regulating the Akt/mTOR and ERK1/2 signaling pathways. PTC isolated from WT and TRPC6^-/-^ mice were treated with H_2_O_2_ (0.5 mM 12 h) or left untreated. **a** Western blot images showing the phosphorylated and total protein expression of Akt, p70S6K, and ERK1/2. Bar graphs shows the relative quantification of p-Akt/Akt, p-p70S6K/p70S6K, and p-ERK/ERK. Data are expressed as mean ± SEM, *n* = 4; *P < 0.05. **b** Representative western blot images are showing the LC3, and the phosphorylated and total protein expression of Akt and ERK1/2 after treatment with H_2_O_2_ in the presence and absence of the Akt inhibitor (MK2206, 5 μM) and the ERK inhibitor (U0126, 25 μM). **c** Representative western blot images of LC3 in primary PTC isolated from WT and TRPC6^-/-^ mice after treatment with H_2_O_2_ in the presence and absence of MK2206 (5 μM) and U0126 (25 μM)

mTOR kinase is likely the core regulator of autophagy^49^. It has been demonstrated that ROS affects autophagy through the inhibition of the Akt/mTOR pathway^35^. Additionally, previous studies have suggested that H_2_O_2_ treatment causes the activation of ERK1/2, which regulates autophagy in many cell types. We postulated that an Akt/mTOR-related or ERK-related signal response could be activated in PTC upon oxidative stress. As expected, we found that H_2_O_2_ treatment increased phosphorylation of Akt (Ser473), mTOR (Ser2448) and ERK1/2. Primary PTC from TRPC6^-/-^ mice showed lower levels of p-Akt and p-ERK1/2 than their WT counterparts (Fig. 7a). Therefore, we speculate that oxidative stress triggered TRPC6-Ca^2+^ signaling to phosphorylate Akt and ERK, thereby inhibiting autophagy and promoting cell apoptosis.

To further prove the signaling pathways involved in autophagy regulation, we treated primary PTC with H_2_O_2_ in the presence and absence of the selective blockers of Akt (MK2206) and ERK (U0126). Western blot results showed that 5 μM MK2206 and 25 μM U0126 dramatically blocked the phosphorylation of Akt and ERK, respectively, thereby increasing LC3-II expression in both control and H_2_O_2_-treated PTC (Fig. 7b). Furthermore, TRPC6 knockout increases LC3-II expression in H_2_O_2_-treated PTC, similar to MK2206 and U0126 (Fig. 7c). Accordingly, these data reveal that the PI3K/Akt/mTOR and ERK1/2 pathways are indeed involved in ROS/TRPC6-mediated autophagy inhibition.

## Discussion

In the present study, we observed that TRPC6 knockout significantly increased autophagic flux and decreased the apoptosis rate in PTC upon oxidative stress. Additionally, autophagy blockage promoted H_2_O_2_-induced PTC apoptosis, representing cross talk between autophagy and apoptosis in PTC. Moreover, we demonstrated that TRPC6 inhibited autophagic flux and aggravated oxidative stress-induced damage in PTC by positively regulating the PI3K/Akt/mTOR and Ras/Raf/ERK signaling pathways.

TRPC6 is expressed in the renal epithelial cells of different tubule segments (the proximal tubule, Henle’s loop, distal tubule, and collecting duct) and regulates water and solute transport. In the case of kidney oxidative stress, TRPC6 is extensively expressed and plays pivotal roles. Notably, TRPC6 works as a downstream effector of ROS^14,15,50^, and inhibition of ROS activity by N-acetyl-L-cysteine (NAC) eliminates H_2_O_2_-induced TRPC6 expression^50^. It is still unknown, however, whether TRPC6 delivers pro-survival or pro-death signals in PTC upon oxidative stress. A previous study by our group demonstrated that TRPC6 mediates excessive calcium entry and plays a detrimental role in diabetic nephropathy-induced podocyte injury^43^. We also reported that TRPC3- and TRPC6-mediated Ca^2+^ entry triggers cell death upon I/R injury of cardiomyocytes in the heart^41^ and astrocytes in the brain^42^, supporting the detrimental role of TRPC6 in I/R injury. However, since different organs have different physiological and pathological characteristics, the exact role of TRPC6 in renal oxidative stress injury is needed to be further studied. In this study, we show that the inhibition of TRPC6 activates autophagy and attenuates PTC apoptosis upon oxidative stress.

It is conceivable that autophagy is upregulated and plays an important role in oxidative stress injury. Disruption of autophagic flux has been reported to aggravate oxidative stress-induced tubule damage^24–27^. Jiang et al.^24^ reported that proximal tubule-specific Atg7 knockout mice exhibited increased renal injury compared with wild-type mice upon I/R injury. Highly metabolically active PTC are more vulnerable and susceptible to ischemic conditions and suffer the most severe injury upon oxidative stress, which leads to PTC damage and apoptosis^3–5^. PTC are particularly dependent on autophagy to maintain homeostasis and respond to oxidative stress^18^. Intracellular Ca^2+^ is an important regulator of autophagy^51–54^, and TRPC6 is a widely expressed nonselective calcium-permeable cation channel that is a major factor for calcium entry in nonexcitable cells. In 2016, Ma et al.^15^ reported that TRPC6 was sensitive to redox, and ROS-induced renal damages were partly due to modulating TRPC6/Ca^2+^ signaling. Therefore, we studied the effect of TRPC6 on regulation of autophagy in PTC. Our result showed that PTC isolated from TRPC6^-/-^ mice exhibited higher levels of autophagy compared with PTC from WT mice. Additionally, we, for the first time, demonstrate that the inhibition of TRPC6 promotes autophagic flux and ameliorates H_2_O_2_-induced apoptosis of PTC.

In 2015, Yu et al.^55^ reported that Ang II activates autophagy in podocyte and that silencing TRPC6 could stabilize autophagy induced by Ang II. Recently, Gao et al.^56^ demonstrated that Ang II could increase TRPC6-mediated Ca^2+^ influx and enhance autophagy in podocytes. These data, in contrast to ours, showed an activating effect of TRPC6 on autophagy in podocytes. This could be due to the different cell types, as well as the source of TRPC6-mediated Ca^2+^ entry (SOCE or ROCE). Our study suggests that TRPC6-mediated SOCE increases intracellular Ca^2+^ in PTC, activates mTOR and ERK, and thus inhibits autophagic flux. Studies have shown that Tg, an endoplasmic reticulum Ca^2+^ mobilizing agent, inhibits both basal and starvation-induced autophagy by blocking autophagosomal fusion with the endocytic system^54,57^. Autophagic flux has also been shown to be inhibited by Ca^2+^ entering via SOCE in acute pancreatitis^58^, which leads to vacuolization of the pancreatic acinar cells. Our data not only support these studies, but also identify that Ca^2+^ entry via TRPC6 is essential in autophagy regulation by SOCE.

PI3Ks are a family of enzymes and have been categorized into three classes: class I, II, and III. Class I PI3K catalyzes its substrate, PtdIns(4,5)P2, to produce PtdIns(3,4,5)P3, which then triggers the downstream signaling Akt activation. Activated Akt eventually leads to the activation of mTOR complex I that then inhibits autophagy. In contrast, class III PI3K complexes with Beclin1 and ATG14 and participates in phagophore formation to promote autophagy. Here we focused on the regulatory effect of TRPC6 on PI3K class I signaling in renal oxidative stress injury. Although it is well-acknowledged that the PI3K/Akt pathway directly mediates anti-death and pro-survival effects, it has also been reported to promote cell death^32,33^. In this study, we demonstrate that H_2_O_2_ induces upregulation of TRPC6 in PTC. The overexpression of TRPC6 increases the detrimental intracellular Ca^2+^ concentration, which, in turn, activates the PI3K/Akt/mTOR pathway, leading to Akt phosphorylation, mTOR activation, and autophagy inhibition.

The mitogen-activated protein kinases (MAPKs) have been classified into three major subfamilies: the extracellular signal-regulated kinase (ERK), the c-Jun N-terminal kinase (JNK), and the p38 kinase. Previous studies have suggested that H_2_O_2_ treatment caused the activation of ERK1/2 and that PD98059, an inhibitor of ERK1/2 upstream kinase MEK1/2, reduced H_2_O_2_-induced cell death^59–61^. However, it is still unclear how the ERK1/2 pathway was affected upon H_2_O_2_ treatment. In this study, we emphasized that H_2_O_2_ induced ROS generation and TRPC6 overexpression, thus leading to the increase of intracellular calcium and persistent ERK1/2 activation. The relationship between ERK1/2 pathway and autophagy is unclear. Activation of ERK1/2 is generally thought to confer a promoting effect on autophagy. Conversely, sustained activation of the ERK1/2 pathway inhibits autophagy at the maturation step by promoting the formation of large defective autolysosomes and commits the cell to autophagic vacuolation^39,40,62^. In the present study, we observed that TRPC6-mediated calcium entry led to persistent activation of ERK1/2 and contributed to the inhibition of autophagic flux.

It has also been shown previously that oxidative stress triggers TRPM2-mediated Ca^2+^ influx to inhibit the induction of autophagy via CAMK2-BECN1 signaling^63^. He et al.^64^ reported that Ca^2+^/Calcineurin suppresses AMPK-dependent cytoprotective autophagy in cardiomyocytes under oxidative stress. In this study, we demonstrated that oxidative stress activates TRPC6-induced SOCE to inhibit autophagy and thus causes PTC to become more susceptible to damage. Despite that the specific mechanism underlying oxidative stress-mediated autophagy inhibition was unclear, we speculate that the autophagosomal fusion with autolysosomes, as well as the autophagic vesicle degradation and recycling may be involved.

Collectively, our results reveal a novel role for TRPC6 in the mechanism of autophagy regulation in PTC. We demonstrate that the inhibition of TRPC6 either by genetic deletion or pharmacological blockade enhances reno-protective autophagy by negatively modulating PI3K/Akt/mTOR and Ras/Raf/ERK signaling pathways and attenuating H_2_O_2_-induced apoptosis in PTC. Furthermore, autophagy blockage prevents the protective effect of TRPC6 inhibition or knockout on H_2_O_2_-induced PTC apoptosis. Data from this study provide novel insight into the intricate connections that link the ROS/TRPC6/Ca^2+^ pathway with cell death via modulation of autophagy. Moreover, our data are important for understanding the effects of TRPC6 on ROS-mediated autophagy and the cross talk between autophagy and apoptosis in PTC. Furthermore, TRPC6 may become a new therapeutic target of renal oxidative stress injury in the future.

## Materials and methods

### Mice

TRPC6-deficient (TRPC6^-/-^) mice on a 129SvEv background were generated at the Comparative Medicine Branch (CMB) of the National Institute of Environmental Health Sciences (NIEHS), North Carolina, USA^65^. WT 129SvEv mice were also introduced from NIEHS and served as controls for the KO mice. Age-matched male KO and WT controls were used for all studies. Animals were treated in compliance with the Guide for the Care and Use of Laboratory Animals (National Academy of Science). Animals were kept on a 12-h light–dark cycle in a temperature-controlled room with ad libitum access to food and water. All animal studies were approved by the Animal Care and Utilization Committee of Huazhong University of Science and Technology.

### Primary culture of mouse renal proximal tubular cells

Primary PTC were extracted from male mice (21–30 days) under sterile conditions according to previously described methods^66^. Mice were sacrificed by cervical dislocation, and kidneys were harvested and immediately transferred to cold D-Hanks Balanced Salt Solution (DS) with 1% penicillin–streptomycin (Life Technologies, Grand Island, N.Y., USA). After the renal capsule was removed, the cortical tissue, carefully separated from the medulla, was finely minced, washed twice, and digested with collagenase (DS with 0.1% (wt/vol) type-2 collagenase) (Worthington Biochemical Corporation, LS004176, USA) in a shaking incubator at 37 °C for 10 min for 4 times. After digestion, the supernatant was passed through two nylon sieves (pore size 180 μm and 75 μm, Bio-Swamp, c1842, CHN). The fragments that remained in the 75-μm sieve were resuspended with DS. Then the suspension was washed with DS twice and resuspended into the appropriate amount of culture medium: 1:1 DMEM/F12 (Hyclone, SH30023.01B, USA) supplemented with 1% fetal bovine serum (FBS) (Serapro, S601S, GER), HEPES 15 mM, L-glutamine 2.5 mM, insulin 10 μg/ml, transferrin 5.5 μg/ml, selenium 5 μg/ml (ITS, sigma, I3146, USA), sodium pyruvate 0.55 mM (Bio-Swamp, c1809, CHN), nonessential amino acids 10 mM (HyClone, SH30238.01, USA), penicillin 100 IU/ml, and streptomycin 100 μg/ml, buffered to pH 7.4 and an osmolality of 325 mosmol/kgH_2_O. The tubule fragments were seeded onto polylysine-coated glass slides and left unstirred for 72 h at 37 °C and 95% air–5% CO_2_ in a standard humidified incubator (Thermo Fisher Scientific, USA). Culture medium was replaced initially at 72 h and every 2 days subsequently. After 5–7 days, cell cultures were organized as a confluent monolayer.

### Antibodies and reagents

The primary antibodies against Akt (9272), p-Akt (Ser473) (4060 P), cleaved caspase-3 (9661), p-p70S6K (9205), p70S6K (9202), p-ERK1/2 (4370), and ERK1/2 (4695) were purchased from Cell Signaling Technology. The primary antibodies against TRPC3 (ACC-016) and TRPC6 (ACC-017) were purchased from Alomone. The primary antibody against LC3B (L7543) was purchased from Sigma-Aldrich. The primary antibody against β-actin (TA-09) was purchased from the ZhongShan Biotechnology Company. The secondary antibodies HRP-conjugated anti-rabbit IgG (110777) and anti-mouse IgG (117228) were purchased from the KeRui Biotechnology Company. H_2_O_2_ (107298) was purchased from Millipore. Chloroquine (C6628) and t-BOOH (458139) were purchased from Sigma-Aldrich. SAR7334 (HY-15699), bafilomycin A1 (HY-100558), MK2206 (HY-10358), and U0126 (HY-12031) were purchased from MedChemExpress.

### Cell culture and treatment

HK-2 cell line (a permanent and well-characterized human proximal tubular cell line) was purchased from ATCC and cultured in DMEM/F12 supplemented with 10% FBS, 100 IU/ml penicillin, and 100 µg/ml streptomycin. Culture medium was replaced every 2 days. For H_2_O_2_ treatment, HK-2 cells and primary PTC were stimulated with 0.5 mM H_2_O_2_ diluted in serum-free medium for 12 h to mimic oxidative stress injury of proximal tubular cells in vitro.

### Transmission electron microscopy

Electron microscopy was conducted in the Research Center for Medicine and Structural Biology at Wuhan University according to the standard procedures. In brief, WT and TRPC6^-/-^ mice were sacrificed and primary PTC were isolated for electron microscopy analysis. Cells were scraped and then pelleted by centrifugation at 1000 × g for 15 min at 4 °C, followed by fixation for 24 h at 4 °C in 2.5% glutaraldehyde in 0.01 M PBS (NaCl 137 mM, KCl 2.7 mM, Na_2_HPO_4_ 81 mM, KH_2_PO_4_ 1.4 mM, pH 7.4). According to the procedure, samples were dehydrated and embedded in Embed-812 resin. Then, 60–70-nm sections were cut using an ultramicrotome (EMVC7/Leica, GER) and stained with uranyl acetate and lead citrate. Finally, autophagic vacuoles were observed with a transmission electron microscope (TEM, Hitachi, Japan).

### Cell viability assay

PTC were seeded in 96-well plates, with 3000 cells per well incubated with 0.5 mM H_2_O_2_ for different times in the presence and absence of SAR7334. Cell viability was assessed by CCK-8 (Cell Counting Kit, ZOMANBIO, ZP328, CHN) according to the manufacturer’s protocol. The optical density (OD) was measured at 450 nm.

### LDH assay

After H_2_O_2_ treatment, the cell culture medium at different time points was transferred to 96-well plates. Total cell death was measured by the release of lactate dehydrogenase (LDH) from cells to the culture medium. Apoptosis of primary PTC was determined with LDH release using a LDH assay kit (Nanjing Jiancheng Bioengineering Institute, CHN), according to the manufacturer’s protocol. The optical density (OD value) was measured at 450 nm.

### Measurement of mitochondrial membrane potential

To measure mitochondrial membrane potential **(**ψm**)**, primary PTC were exposed to H_2_O_2_ (0.5 mM 12 h) in the absence and presence of TRPC6 inhibitor SAR7334 (100 nM). Cells were washed with PBS and incubated with 5 µM JC-1 dye (Bio-Swamp, CHN) at 37 °C for 20 min in the dark. After incubation with the dye, the plates were washed 3 times with PBS. Fluorescence was observed first at an emission wave length of 595 nm (red) and then at an emission wave length of 529 nm (green) under a laser scanning confocal microscope (Olympus FV3000, Japan). The percentage of mPT-positive PTC was calculated to quantify changes in mitochondrial membrane potential.

### Tandem mRFP-GFP-LC3 fluorescence microscopy

Autophagic flux was tested by transfecting tandem mRFP-GFP-LC3 plasmid and observing the green and red signal by fluorescence microscopy. It shows green and red before the fusion of autophagosome with lysosome and exhibits only red after fusion, since the acidic environment of lysosomes causes the quenching of green fluorescence^48^. After 24 h of transfection, HK-2 cells were plated onto glass slides. On the following day, the cells were treated with 0.5 mM H_2_O_2_ in the absence and presence of SAR7334 (100 nM) and BAF (20 nM) for 12 h. After incubation, cells were fixed with 4% paraformaldehyde for 15 min and rinsed with PBS twice. Cells were mounted and visualized under a confocal microscope. To quantify the autophagy level, six different confocal microscopy images were randomly chosen and the yellow and red dots, which represent autophagosomes and autolysosomes^48^, were examined.

### Flow cytometric apoptosis assay

Apoptosis was assessed by flow cytometry analysis. Primary PTC were stained with fluorescein isothiocyanate-conjugated annexin-V protein (Annexin V) and propidium iodide (PI) using an AnnexinV/PI apoptosis kit (MultiSciences Biotech Co., CHN). Briefly, cells of different groups were collected at a concentration of 1 × 10^5^ cells/ml, mixed with AnnexinV-FITC and PI according to manufacturer’s recommendation, and analyzed using a flow cytometer. Data were analyzed by the Cell Quest software (BD Biosciences, USA).

### TUNEL assay

DNA damages of primary PTC were detected and analyzed by terminal deoxynucleotidyl transferase (TdT) dUTP nick end labeling (TUNEL) method using a commercially available kit (In Situ Cell Death Detection Kit, Roche, USA). Briefly, after H_2_O_2_ treatment (0.5 mM 12 h), cells on the slides were fixed with 4% paraformaldehyde for 1 h, blocked with 3% H_2_O_2_ in methanol, and permeabilized with 0.1% (v/v) Triton X-100 for 2 min on ice. Samples were then incubated in 50 µl TUNEL reaction mixture for 1 h at 37 °C in a dark and humidified atmosphere. Nuclei were stained with 1 µg/ml DAPI (Roche, USA) for 10 min. Positive TUNEL staining was observed under a confocal microscope. The TUNEL index was determined by counting the positive and negative stained PTC in each of the six fields of vision.

### Plasmid transfection and lentiviral infection

The plasmids pcDNA3-TRPC6 and pcDNA3-EV were described previously^67^. Cells were transfected with the plasmids using the Lipofectamine® 2000 Transfection Reagent (Invitrogen, USA) according to the manufacturer’s protocol. The Opti-MEM (Gibco, USA) medium was replaced with DMEM/F12 and 10% FBS after 6–8 h incubation, and the cells were used for the experiments after 24 h. The shRNA against TRPC6 was from the MISSION^(TM)^ shRNA Library (Sigma-Aldrich). The sequence was as follows: TRPC6, CCGGCCAGAGCATCATTGACGCAAACTCGAGTTTGCGTCAATGATGCTCTGGTTTTTG. ShMOCK refers to an empty vector. Lentivirus production and concentration were done as described^68^. In brief, HEK293T cells were co-transfected with lentiviral vector plasmid (pLKO.1-shTRPC6) and packaging plasmids psPAX2 and pMD2.G, using the PolyJet Transfection Reagent (SignaGen Laboratories, USA). The medium was changed on the next day, and cells were cultured for another 24 h. Conditioned medium was then collected, filtered through a 0.45-μm filter, and concentrated by ultrafiltration using Amicon Ultra filtration units (Millipore, USA). HK-2 cells at 60% confluence were infected with shTRPC6 or shMOCK lentivirus. The medium was replaced 24 h after infection, and then the cells were used for the experiments.

### Calcium imaging

Intracellular Ca^2+^ concentration measurements were obtained from PTC of WT and TRPC6^-/-^ mice preloaded with the Ca^2+^-sensitive fluorescent dye Fura2-AM (Invitrogen, F1201, USA). As described in He et al.^41^, PNAS 2017, briefly, the cells were loaded with 3 μM Fura2-AM in DMEM/F12 1:1 medium for 50 min at room temperature. Then the cells were washed 3 times with HBSS (140 mM NaCl, 5 mM KCl, 10 mM HEPES, 10 mM glucose, and 1 mM MgCl_2_, pH 7.4) medium with 2 mM Ca^2+^ and incubated at room temperature for another 10 min. The coverslips were mounted onto the platform of an inverted epifluorescence microscope. To measure Thapsigargin (Tg, Invitrogen, T7459, USA)-evoked Ca^2+^ entry, cells were bathed in sequence with 50 μM EGTA in HBSS for 3 min, 50 μM EGTA and 2 μM Tg in HBSS for 6 min, and 2 mM Ca^2+^ plus 2 μM Tg in HBSS for 6 min, as shown in the figures. Ca^2+^ entry was also assessed in the absence and presence of the TRPC inhibitor SAR7334. Cytosolic Ca^2+^ was monitored with an Olympus IX51 inverted fluorescence microscope and SlideBook software, using excitation wavelengths of 340 and 380 nm to detect Fura-2/Fura2-Ca^2+^ fluorescence emissions at 510 nm.

### Western blot analysis

Western blot analysis was carried out following standard methods. The cells were lysed with the lysis buffer (50 mM Tris-HCl (pH 6.8), 150 mM NaCl, 1 mM EDTA, 1% NP-40, and 1 mM PMSF) for 40 min on ice. After centrifugation at 12,000 rpm for 15 min at 4 °C, the supernatant was collected. Then the protein sample loading buffer was added and samples were boiled at 95 °C for 10 min. The protein extracts (30 μg) were separated by 8–15% SDS-poly acrylamide gel electrophoresis and transferred to a polyvinylidene difluoride (PVDF) membrane (Roche, USA). The membrane was blocked with 5% (w/v) skim milk in PBS Tween-20 (PBST; 0.05%) for 1 h and then incubated with the primary antibodies (1:1000 in PBST) at 4 °C overnight. Following three washes with PBST, the PVDF membrane was incubated with the appropriate HRP-conjugated secondary antibodies (1:10,000 in PBST) for 1 h at room temperature. The immunoreactive bands were developed with the Pierce ECL (Thermo Fisher Scientific, USA) chemiluminescence reagents. The relative quantity of the ECL-positive proteins was analyzed with the Quantity One software (Bio-Rad, Hercules, CA, USA).

### Statistical analyses

All experiments were performed in triplicate and repeated at least 3 times. The data were expressed as the mean ± standard error of the mean (SEM). The differences across groups were analyzed with one-way variance (ANOVA), and the means of two groups were tested using Student’s *t*- test. Differences were considered statistically significant when *P* < 0.05.

## Electronic supplementary material

Supplementary Figure Legends

Supplementary figure 1

Supplementary figure 2

Supplementary figure 3
