# Supplementary material for: Transient receptor potential channel 6 knockdown prevents apoptosis of renal tubular epithelial cells upon oxidative stress via autophagy activation
Source: Cell Death Dis. 2018 Oct 3;9(10):1015. doi: 10.1038/s41419-018-1052-5 (PMC6170481; doi:10.1038/s41419-018-1052-5)
Supplement: Supplementary file 1 — Supplementary Figure Legends [file 41419_2018_1052_MOESM1_ESM.doc]

**Supplementary Figure Legends**

**Supplementary Figure 1: Oxidative stress increases TRPC6 expression and inhibits cyto-protective autophagy. a** Representative western blot images of TRPC6 and TRPC3 in primary proximal tubular cells (PTC) after treated with different concentrations of t-BOOH for 12 h. Data are shown as mean ± SEM, n = 3; NS indicates not significant, **P* < 0.05. **b** Representative western blot images of LC3 in primary PTC after treated with different concentrations of t-BOOH for 6 h and 12 h. Data are shown as mean ± SEM, n = 3; **P* < 0.05. **c** Representative western blot images of LC3 in primary PTC after treated with different concentrations of H_2_O_2_ for 6 h and 12 h. Data are shown as mean ± SEM, n = 3; **P* < 0.05. **d** Representative western blot images of LC3 and CC3 in primary PTC from WT and TRPC6^-/-^ mice after treated with t-BOOH (0.5 mM 6 h). Data are shown as mean ± SEM, n = 3; **P* < 0.05.

**Supplementary Figure 2: TRPC6 knock out decreases store-operated calcium entry (SOCE) in PTC.** Representative traces showing the transient increase in [Ca^2+^]_i_ in PTC isolated from WT or TRPC6^-/-^ mice. Summary of peak SOCE values are shown as mean ± SEM, n = 3 (40-50 cells per experiment); **P* < 0.05.

**Supplementary Figure 3: Identification of the efficiency of TRPC6 overexpression or knock down. a** Semi-quantitative immunoblotting and mRNA expression studies demonstrating the expression of TRPC6 after sh-TRPC6 or sh-MOCK lentivirus infection in HK-2 cells. Data are shown as mean ± SEM, n = 3; **P* < 0.05. **b** Semi-quantitative immunoblotting and mRNA expression studies demonstrating the expression of TRPC6 after pcDNA3-TRPC6 or pcDNA3-EV infection in HK-2 cells. Data are shown as mean ± SEM, n = 3; **P* < 0.05.
