# Supplementary figures and images for: Transient receptor potential channel 6 knockdown prevents apoptosis of renal tubular epithelial cells upon oxidative stress via autophagy activation
Source: Cell Death Dis. 2018 Oct 3;9(10):1015. doi: 10.1038/s41419-018-1052-5 (PMC6170481; doi:10.1038/s41419-018-1052-5)

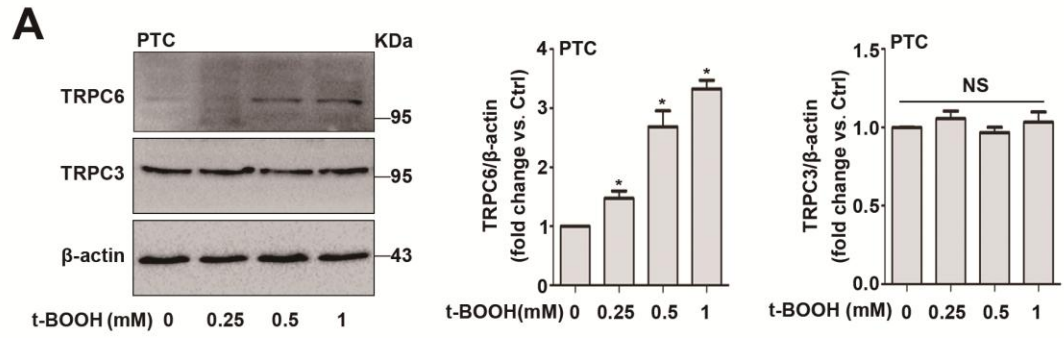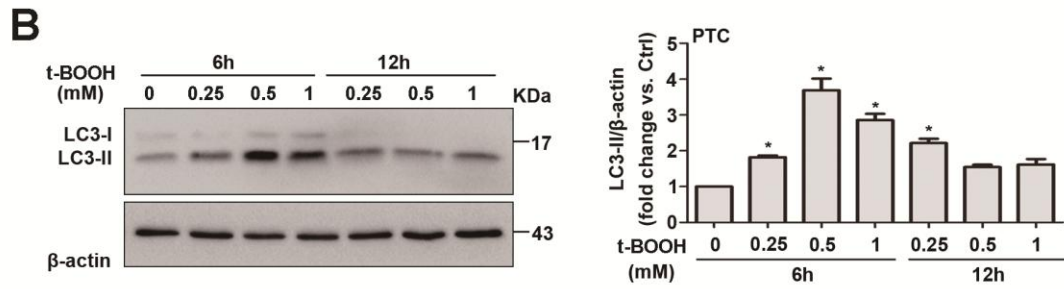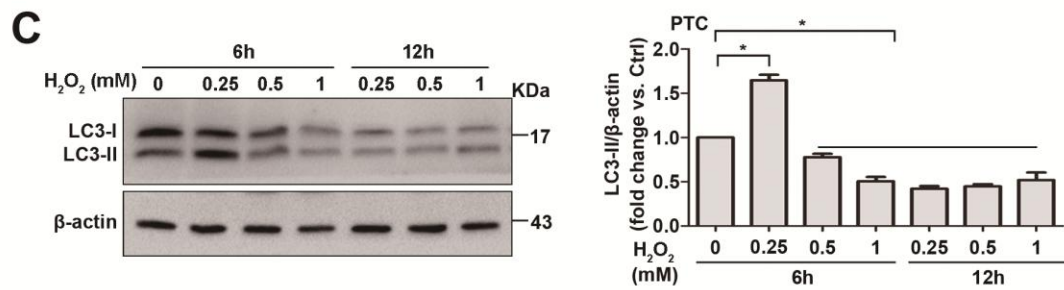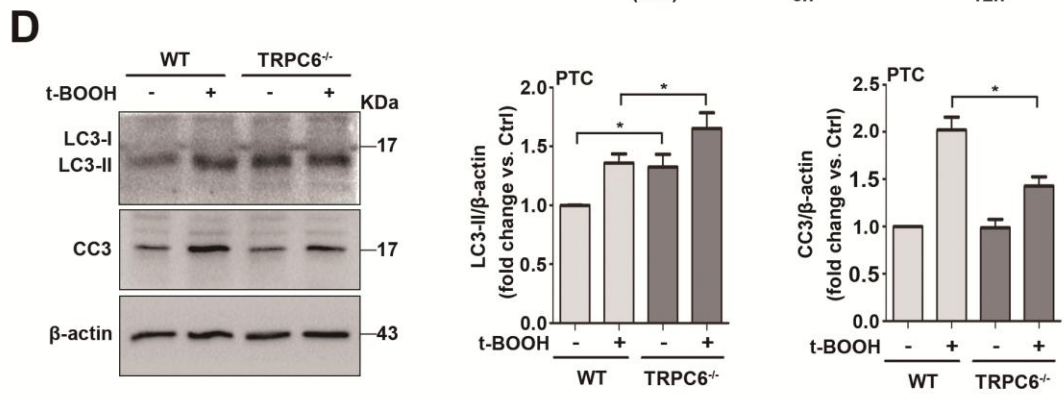

Supplement: Supplementary file 2 — Supplementary figure 1 [file 41419_2018_1052_MOESM2_ESM.pdf]

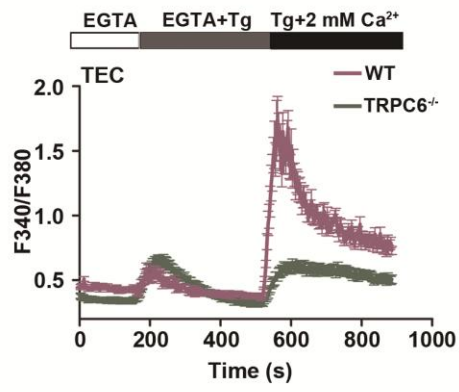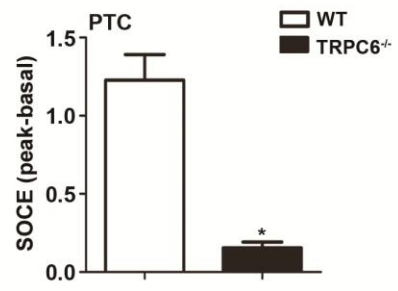

Supplement: Supplementary file 3 — Supplementary figure 2 [file 41419_2018_1052_MOESM3_ESM.pdf]

**A**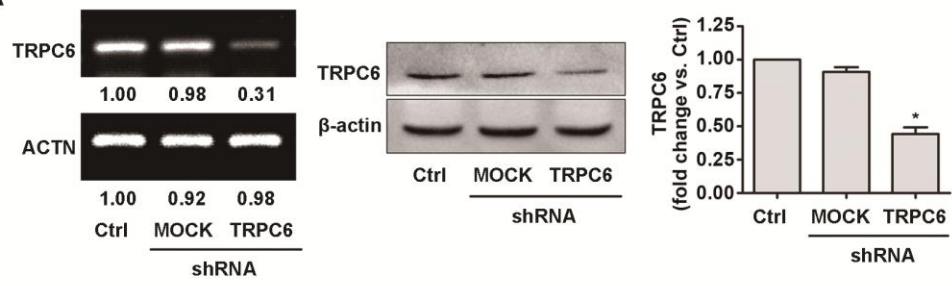**B**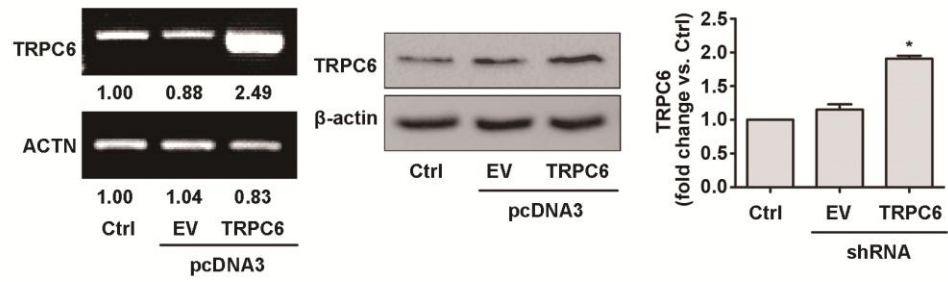

Supplement: Supplementary file 4 — Supplementary figure 3 [file 41419_2018_1052_MOESM4_ESM.pdf]
